# Access to novel drugs and therapeutics for children and youth: Eliciting citizens' values to inform public funding decisions

**DOI:** 10.1111/hex.13697

**Published:** 2023-01-14

**Authors:** Cindy L. Gauvreau, Lisa Wight, Mathushan Subasri, Antonia Palmer, Robin Hayeems, Alysha Croker, Julia Abelson, Brent Fraser, Yvonne Bombard, Charlotte Moore Hepburn, Michael G. Wilson, Avram Denburg

**Affiliations:** ^1^ Child Health Evaluative Sciences Program The Hospital for Sick Children Research Institute Toronto Ontario Canada; ^2^ Ac2orn: Advocacy for Canadian Childhood Oncology Research Toronto Ontario Canada; ^3^ Institute for Health Policy, Management and Evaluation University of Toronto Toronto Ontario Canada; ^4^ Centre for Policy, Pediatrics and International Collaboration Health Products and Food Branch, Health Canada Ottawa Ontario Canada; ^5^ Health Research Methods, Evidence and Impact, Faculty of Health Sciences McMaster University Hamilton Ontario Canada; ^6^ Pharmaceutical Reviews, CADTH Ottawa Ontario Canada; ^7^ Ontario Institute of Cancer Research Toronto Ontario Canada; ^8^ Li Ka Shing Knowledge Institute, St. Michael's Hospital, Unity Health Toronto Ontario Canada; ^9^ Pediatric Medicine The Hospital for Sick Children Toronto Ontario Canada; ^10^ McMaster Health Forum, Health Evidence and Impact McMaster University Hamilton Ontario Canada; ^11^ Present address: Master of Public Health University of British Columbia Vancouver British Columbia Canada; ^12^ Present address: School of Medicine, Faculty of Medicine and Health Sciences McGill University Montreal Quebec Canada

**Keywords:** access to novel drugs, children and youth, citizens' values, deliberative public engagement, health technology assessment, public funding decisions

## Abstract

**Introduction:**

The unique evidentiary, economic and ethical challenges associated with health technology assessment (HTA) of precision therapies limit access to novel drugs and therapeutics for children and youth, for whom such challenges are amplified. We elicited citizens' perspectives about values‐based criteria relevant to the assessment of paediatric precision therapies to inform the development of a child‐tailored HTA framework.

**Methods:**

We held four citizen panels virtually in May–June 2021, informed by a plain‐language citizen brief summarizing global and local evidence about the challenges, policy and programmatic options and implementation strategies related to enhancing access to precision therapies for Canadian children and youth. Panellists were recruited through a nationally representative database, medical/patient networks and social media. We inductively coded and thematically analysed panel transcripts to generate themes and identify priority values.

**Results:**

The perspectives of panellists (*n* = 45) coalesced into four overlapping themes, with attendant subthemes, relevant to a child‐tailored HTA framework: (1) Childhood Distinctions: vulnerability, ‘fair innings’, future potential, family impacts; (2) Voice: agency of children and youth; lived versus no lived experience; (3) One versus Many: disease severity, rarity, equity, unmet need and (4) Health System Governance: funding, implementation inequities, effectiveness and safety. Participants broadly agreed that childhood distinctions, particularly family impacts, justify child‐tailored HTA. Dissent arose over whose voice should inform HTA and how such perspectives are best incorporated.

**Conclusions:**

Citizens can offer unique insights into criteria relevant to the development or revision of HTA frameworks to capture holistic, societally responsive dimensions of value attached to unique contexts or populations, including children. Balancing the hopes and expectations of patients and caregivers for access to expensive but potential life‐altering therapies against the opportunity costs borne by encompassing health systems is a fundamental challenge that will require rigorous methods to elicit, weigh and reconcile varied views.

**Patient or Public Contribution:**

A patient advocate served on the steering committee of this study and co‐authored this article. Key informants for the Citizen Brief included patient advocates and caregivers; a separate patient advocate reviewed the Brief before dissemination. Qualitative and quantitative data were collected from the general public and caregivers of children, with written consent.

## 1 INTRODUCTION

The genomic revolution poses challenges to the responsible stewardship of limited healthcare resources and the equitable distribution of benefits. Precision therapies, designed to target patient‐level genetic differences that drive disease development and behaviour, hold promise for patients suffering from rare or hard‐to‐treat diseases, which may lack effective alternative treatment options.^1^, ^2^ However, access to and uptake of these therapies is restricted by limited data on clinical effectiveness and by their typically large price tags, posing challenges for traditional health technology assessment (HTA).^3^, ^4^, ^5^ Health systems have yet to develop assessment standards and policies to address the unique evidentiary, economic and ethical challenges presented by precision therapies, constraining the incorporation of best evidence and patient and public values into HTA processes and, by extension, public funding decisions.^4^, ^6^, ^7^, ^8^, ^9^

Children who suffer from rare and complex diseases may have the most to gain from precision health technologies, yet they are being left behind at multiple levels of therapy development and access. First, clinical trials in children are limited by small population sizes, complexities of trial enrolment and weak industry interest.^10^ Second, submissions to HTA bodies seldom include evidence for paediatric indications, meaning funding recommendations are routinely based on and intended for adult indications. Third, current funding assessments rarely incorporate the unique sociobiology of child health and illness.^11^, ^12^, ^13^, ^14^ Governments and researchers are responding to the first two constraints by developing unique trial designs, creating disease networks, enacting legislation to compel and incentivize pharmaceutical companies to generate paediatric evidence and formulating policy targeting funding for orphan and rare diseases.^15^, ^16^, ^17^, ^18^, ^19^, ^20^, ^21^, ^22^

The third constraint remains largely unaddressed and stems from a more fundamental problem: the lack of HTA approaches that attend to the unique dimensions of child health and illness.^12^, ^23^, ^24^, ^25^, ^26^ HTA frameworks set out the principles and methods for systematic weighing of clinical, economic, organizational and patient‐related impacts of novel interventions to ascertain value increments compared to existing alternatives, with the extra charge of assessing contextual equity, sustainability and health system implementation impacts. In the context of precision health interventions, evaluations must weigh uncertainties related to limited data on safety and efficacy. When applied to child health technologies, these frameworks face the additional challenge of incorporating the unique evidentiary, physiological, health‐system and societal considerations attached to children.^27^, ^28^, ^29^, ^30^, ^31^ The absence of a standardized framework for the evaluation and reimbursement of precision health technologies for children thus has significant consequences for children and youth.

Effective citizen engagement and public deliberation have been shown to provide rich and nuanced insights on issues of societal importance.^32^, ^33^, ^34^, ^35^, ^36^ Structured and meaningful incorporation of these insights into health policy‐making processes helps to legitimize decisions that impact the populace. In many jurisdictions, including Canada, public engagement has been embraced as a component of HTA deliberations, but implementation is variable and best practices for evidence‐informed deliberation are still evolving.^37^, ^38^, ^39^ Furthermore, input from *non‐patient publics*, particularly youth, is often neglected or underrepresented in HTA processes, which might erode the societal legitimacy of recommendations provided or decisions made. We conducted a series of citizen panels with members of the Canadian public to elicit and prioritize values relevant to HTA for precision child health technologies. This research constituted the initial phase in the development of a value assessment framework for precision therapies for children and youth explicitly informed by societal values.

## 2 METHODS

### 2.1 Study design

We convened four citizen panels, based on an established approach developed by the McMaster Health Forum,^40^ to identify public values related to HTA for precision child health technologies. The panels were informed by an evidence package and were convened virtually using Microsoft Teams™ between April and June 2021, each lasting 3 h.

An interdisciplinary steering committee provided expert guidance and oversight for the project. The committee consisted of academic paediatricians, a bioethicist, representatives from Health Canada and the Canadian Agency for Drugs and Technology in Health (CADTH), a patient advocate and health services researchers with expertise in genomics, HTA, health systems and policy analysis, deliberative processes and qualitative research methods. The steering committee met six times between November 2020 and September 2021, and was given opportunities to review all materials.

### 2.2 Evidence package

To support informed deliberations, we provided panellists at least 1 week before the panels an evidence package in the form of a citizen brief and complementary videos. We compiled evidence from systematic searches of online evidence synthesis repositories (e.g., Health Systems Evidence) and medical literature databases, and supplemented it with grey literature identified through targeted searches of relevant regulatory and HTA institutional websites. The citizen brief summarized relevant research on the following: (1) context about why precision therapies for children and youth in Canada requires public input; (2) key challenges that exist for accessing precision therapies for children and youth; (3) three elements of a potential comprehensive approach for addressing these challenges and (4) implementation considerations.^41^ We presented the evidence in plain language and also conveyed it using a mix of figures, infographics and case studies. We also posed questions for panellists to consider, including those focussed on a child‐tailored HTA, that would be used to guide deliberations.

Input from key informants (*n* = 17) helped shape the focus and content of the brief. The key informants included paediatric specialists in rare and chronic conditions, policymakers, leaders of patient advocacy groups, health researchers, industry professionals, representatives from CADTH and Quebec's Institut national d'excellence en santé et services sociaux (INESSS), and regulatory leaders from Health Canada, the FDA and the European Medicines Agency. Four merit reviewers, comprising a health economist, two policymakers and a patient/citizen representative, critically assessed the accuracy, relevance and readability of the brief.

The complementary videos provided general lay information on precision medicine and HTA processes and were publicly available through YouTube™. (Links are found in Supporting Information: Appendix 1, ‘Complementary Videos’.)

### 2.3 Recruitment and panel composition

As a standard for deliberative public engagements (DPEs), we aimed to convene for each panel 14–16 members of the general public who were diverse in terms of gender, age, socioeconomic status, ethnocultural background and geographic residence for two of the four panels. We recruited panellists in collaboration with AskingCanadians™, which has a panel of more than 600,000 Canadians who are affiliated with loyalty programmes in Canada and are representative of all Statistics Canada demographic categories.^42^ Through a combination of recruitment through AskingCanadians™ and invitation through clinical and patient networks and social‐media advertisements, we aimed to form two population‐focussed panels, another composed of caregivers of children with chronic and complex diseases and a last with youth participants aged 16–21.

### 2.4 Data collection and analysis

Two expert facilitators (A. D. and M. G. W.) provided panellists with opportunities to learn from the evidence and others' experiences, share existing and newly informed views and make conclusions based on their values and preferences. The discussions in the two larger groups (one general public and the youth group) took place in an initial plenary, followed by a break‐out into two groups to allow freer discussion and concluded with a consolidating plenary where individual groups reported on respective key discussion points. In the two smaller groups, panellists remained together in plenary. Panellists were also encouraged to use the ‘chat’ function. The discussion was guided by questions from the citizen brief, focussing on HTA‐related challenges and pertinent approaches to overcome them. We did not aim for consensus among the panellists but rather to identify common ground, differences of opinion and the values underlying different positions. Audio and ‘chat’ exchanges were recorded on Microsoft Teams™, and research team members also took notes and memos throughout the deliberations.

Additionally, online pre‐ and postdeliberation questionnaires were sent to panellists during each panel asking them to rank 12 HTA‐relevant values, which had been identified based on a literature review and prior research.^12^, ^25^, ^43^, ^44^ These were as follows: effectiveness; disease severity; safety; unmet need; future potential; costs; impacts on families; rarity; vulnerability; citizen values and preferences and child and youth views.

Each citizen panel recording was transcribed verbatim, cleaned and then verified against the audio‐recording. Messages in the ‘chat’ function were included in the data set. Data were anonymized and de‐identified to protect panellist confidentiality and were stored in a secure electronic database accessible only by the research team. Following Braun and Clarke's^45^ thematic analysis method, transcripts were inductively coded using NVivo12. Three team members (C. L. G., L. W. and M. S.) iteratively created a codebook and independently coded transcripts, employing constant comparative methods to further refine codes, identify patterns and generate themes (See Supporting Information: Appendix 2, ‘Codebook’). Independent coding results were compared in two rounds of examination by the team to resolve discordance and ensure fidelity to the codebook and theme generation. Analysis of the virtual ‘chat’ messages, researcher notes and coding memos all contributed to theoretical interpretations and enabled reflexivity.^46^

## 3 RESULTS

### 3.1 Panel and panellist characteristics

Table 1 outlines the sociodemographic characteristics of the panellists (*n* = 45). The two general public panels numbered 7 and 14, respectively, the youths numbered 14 and the caregivers 10. While three of the panels were balanced in terms of the number of female and male participants, the caregiver panel was exclusively female. Panellists were from nine of Canada's 10 provinces, with predominant representation from Ontario and Québec, reflecting their relative population sizes within the country. All those with lived experience of accessing paediatric precision therapies were caregivers.

**Table 1:** Sociodemographic characteristics of panellists, *n* = 45

Characteristics	*n*	%
Sex		
Male	13	29
Female	31	69
Prefer not to disclose	1	2
Age (years)		
16–21	14	31
22–34	7	16
35–49	14	31
50–64	9	20
≥65	1	2
Province of residence		
British Columbia	1	2
Alberta	7	16
Saskatchewan	1	2
Manitoba	1	2
Ontario	18	40
Quebec	10	22
New Brunswick	3	7
Nova Scotia	2	4
Prince Edward Island	2	4
Lived experience		
Yes	12	27
No	33	73
Highest educational level attained		
Elementary school	1	2
High school	9	20
Community college	8	18
Technical school	5	11
Bachelor/postgraduate training/professional degree	22	49
Employment status		
Self‐employed	3	7
Full‐time employed	17	38
Part‐time employed	3	7
Unemployed	4	9
Retired	3	7
Student	12	27
Homemaker	3	7
Income level (Canadian dollars)		
<$20,000	6	13
$20,000–$40,000	8	18
$40,000–$60,000	5	11
$60,000–$80,000	8	18
>$80,000	10	22
Prefer not to disclose	8	18

### 3.2 Perspectives on current approaches to HTA processes for child health technologies

Reflecting on existing core criteria for HTA assessments, panellists broadly agreed that current frameworks do not specifically account for the unique dimensions of child health and illness, nor do they capture family perspectives, generally and specifically for paediatric precision therapies. A range of panellists emphasized that current approaches to measuring effectiveness and adjudicating therapeutic benefit may be inadequate or poorly calibrated to the evolving disease dynamics and treatment paradigms in precision medicine as well as to alternative outcomes that patients and caregivers value. One caregiver of a child with cystic fibrosis asserted that HTA assessment ‘gave [a therapy] a negative recommendation because it only improved lung function by 3 to 5%. So that's what it said on paper, but I've been trying to explain to them that maybe 3 to 5% on paper doesn't look like much, but to me that's my child going to a full year of school … not having to miss, you know, 50‐something days of school that he had missed previously’ (Panellist P4‐9). Similarly, some panellists noted a greater need to situate safety considerations within the context of other values—such as disease severity, hope and risk tolerance. As one noted, ‘I imagine often the families will have a very large say into what kind of risk they're willing to take’ (Panellist P1‐2).

Panellists deliberated about costs from both health system and family perspectives, particularly opportunity costs. Several of them highlighted that the full costs associated with a complex or chronic illness, including those related to immediate and long‐term disease impacts, are not wholly considered in HTA, with outsized implications for child health due to their care needs. One caregiver shared, ‘I have two degrees and I haven't worked since [the two children's] diagnosis because it's a lot of work. So that's lost income for our family and it's also lost tax dollars…’ (Panellist P4‐3). Some panellists also felt that the nonmonetary costs of illness, including lost schooling or decrements in well‐being, need to be included in assessments. Although cost‐effectiveness was only implicitly discussed, a number of panellists grappled with the legitimacy of explicit cost‐effectiveness thresholds, such as the commonly referred to $100,000 per QALY, in the face of lived realities of disease for children and families. One panellist stated, ‘…this idea that you can put a price on my child's life, like my daughter is only worth … $100,000 a year … that, you know, just hurts you inside’ (Panellist P4‐2).

### 3.3 Perspectives on priority values and principles for a child‐tailored HTA framework

In thinking about how to create and operationalize a child‐tailored HTA framework, panellists queried, weighed and debated the individual values important to such a framework, including those for precision therapies for children and youth. Their perspectives coalesced into four overarching and overlapping themes: (1) Childhood Distinctions—characterized by consensus that children have unique attributes justifying child‐tailored HTA; (2) Voice—characterized by disagreement about whose perspectives ought to inform HTA; (3) One versus Many—characterized by the discordance between humanitarian and utilitarian perspectives and (4) Health System Governance—characterized by reflection on structural challenges in equitable access to precision therapies determined by the overarching Canadian health system context.

**Figure 1:**
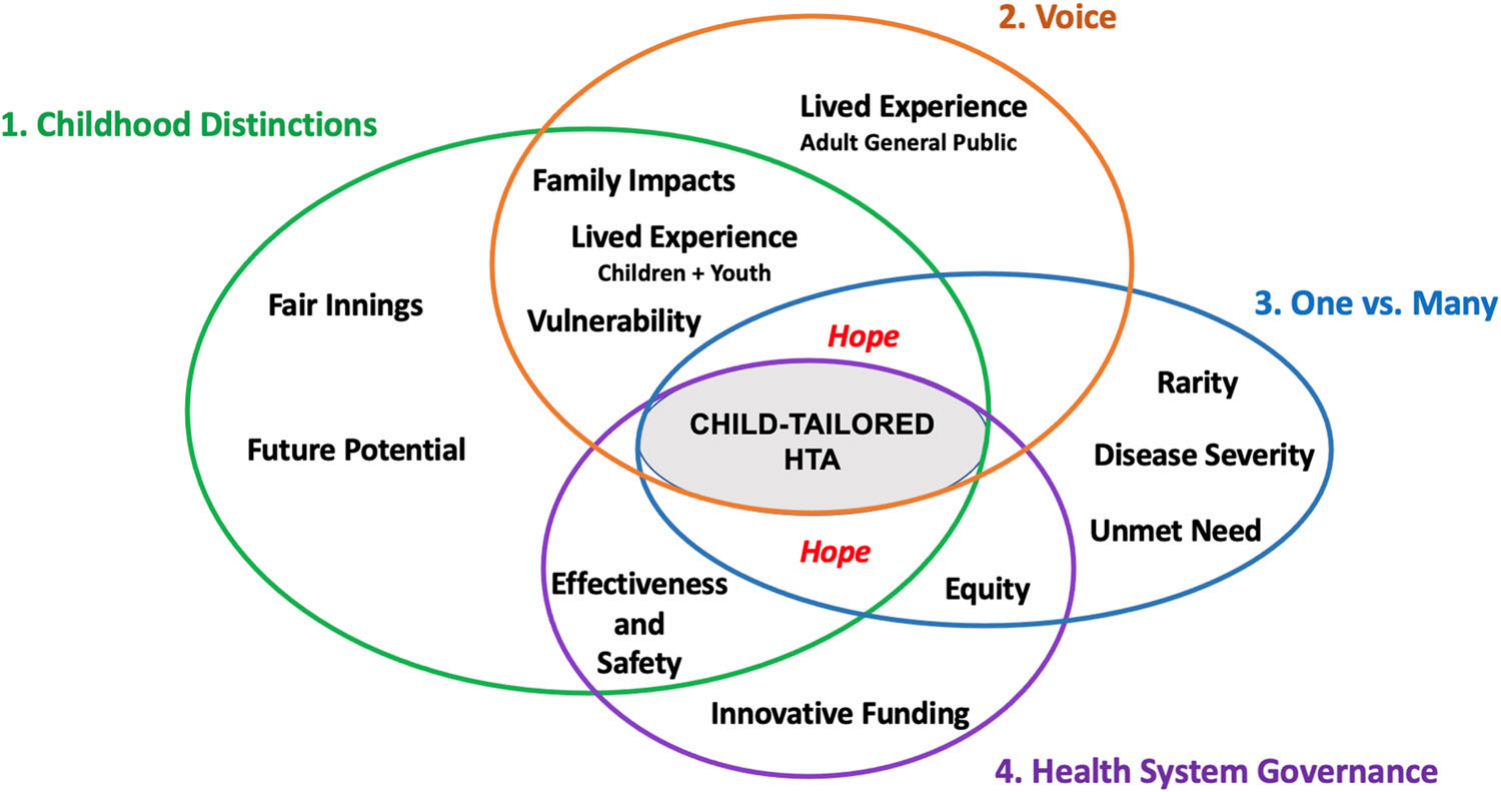
Four overarching themes and subthemes for a child‐tailored health technology assessment (HTA) framework

Figure 1 depicts key interconnections between the overarching themes and subthemes, including ‘Hope’, which was threaded throughout the discussions.

### 3.4 Theme 1: Childhood Distinctions

Childhood Distinctions, a cluster of interrelated phenomena which distinguish children and childhood traits, were paramount in both justifying and framing the development of a child‐tailored HTA framework for most panellists. Discussion focussed on four closely interlinked subthemes that were seen to set children and youth apart from adults: (a) vulnerability; (b) ‘fair innings’; (c) future potential and (d) family impacts.

#### 3.4.1 Vulnerability

A multifaceted concept of childhood vulnerability emerged in panellists' reflections, particularly around the dimensions of physical fragility, cognitive maturity, parental dependence and autonomy within the family. The complex interactions among these dimensions, shaped continuously by age throughout childhood, were reflected in panellists' arguments over how to define, assess and measure vulnerability. The need for proxy voices in medical and policy decision‐making contributed to panellists' conception of childhood vulnerability, and disabilities arising from chronic illnesses were seen to complicate these issues. Panellists agreed that society had a collective responsibility to take protective actions for children, as one succinctly explained: ‘I think society … seems to view children as being more fragile … with how like children [are] first in a lot of rescue stuff … in general with society we kind of try to prioritize children’ (Panellist P3‐2). Nevertheless, panellists sometimes grappled with how to define vulnerability given evolving capabilities and appropriate agency for children. One panellist expressed this ambivalence thus: ‘…making the decisions, it has to be the children when they do have the ability to do so…. I don't know how you would detect the age for that because I think age is really again another debate’ (Panellist P3‐8).

#### 3.4.2 ‘Fair innings’ and future potential

Although these two subthemes were explored discretely, they were often closely linked during discussions. The concept of ‘fair innings’ describes the consideration given to those who have experienced, or are expected to experience, comparatively less of a normal span of healthy life.^47^ Fair innings, panellists felt, constitute the fair entitlement of children to the same healthy longevity as everyone else in society. They argued that it is equitable and justifiable to prioritize resource allocation to children to avert premature death or diminish long‐term physical, cognitive and psycho‐social harms imposed by diseases arising during childhood. Relatedly, children were seen to have more future potential. The future potential was described by panellists in both societal and personal terms, as socioeconomic contributions in future adulthood and as prospective personal growth and fulfilment. Panellists overwhelmingly believed that children ought to be prioritized over adults, observing ‘as much as … you love your grandmother, they've already lived up to their potential … rather than someone who is 10 years old…’ (Panellist P3‐13) and ‘[children] …have a right to have the same amount of time on this Earth as anybody else’ (Panellist P4‐5).

#### 3.4.3 Family impacts

Panellists strongly emphasized that the near‐exclusive reliance of children with complex and chronic illnesses on their families marked their distinction from adults. They emphasized that the impacts of illness routinely spill over to the activities and well‐being of the entire family, including to siblings. Yet, in conventional HTA processes, impacts on the child are evaluated in isolation. Financial costs, such as travel costs for treatment and lost wages, and nonfinancial costs, such as stress and distress, were equally emphasized by panellists. Caregiver panellists most eloquently shared their insights on family impacts: ‘…before my daughter was on the modulator, there was two years where she had a really rough time, sometimes we were at clinic every week and you see five or six different practitioners every time’ (Panellist P4‐3). For this family, and many others, the intensity of treatments for children required an enormous investment of time and energy by all members of the family. Advocacy for their children, whether in the health system to access hard‐to‐obtain therapies or in the policy sphere to raise awareness among decision‐makers, also required substantial effort.

### 3.5 Theme 2: Voice

The issue of which perspectives are most relevant and legitimate as input into funding decision‐making for paediatric precision therapies, and therefore whose voices should be heard in medical and evaluative processes, was the central, and most discordant, theme across all panels. Panellists concurred that the public voice, which they considered distinct from the patient voice, was a key input into funding policies, based on shared beliefs in the universality of the Canadian healthcare system. However, they disagreed on the importance of input from those with lived experience and those without. Some panellists without lived experience deferred to the expertise of those with lived experience. As one panellist shared, ‘I think as a [member of the] general population, if you don't understand the severity, the suffering, … the non‐financial costs to family of dealing with these diseases and the outcomes and the treatment, it's hard to, you know, prioritize these [values] … when you're weighing other possible considerations’ (Panellist P1‐3). Interestingly, caregivers themselves supported including more expansive and removed perspectives in HTA processes, including those without lived experience. Tension was also evident amongst those who thought being a Canadian guaranteed the exercise of voice in a universal healthcare context. Others thought caution was necessary, given misinformation or ignorance about drug regulatory or funding processes, as well as the presence of strong vested interests.

**Figure 2:**
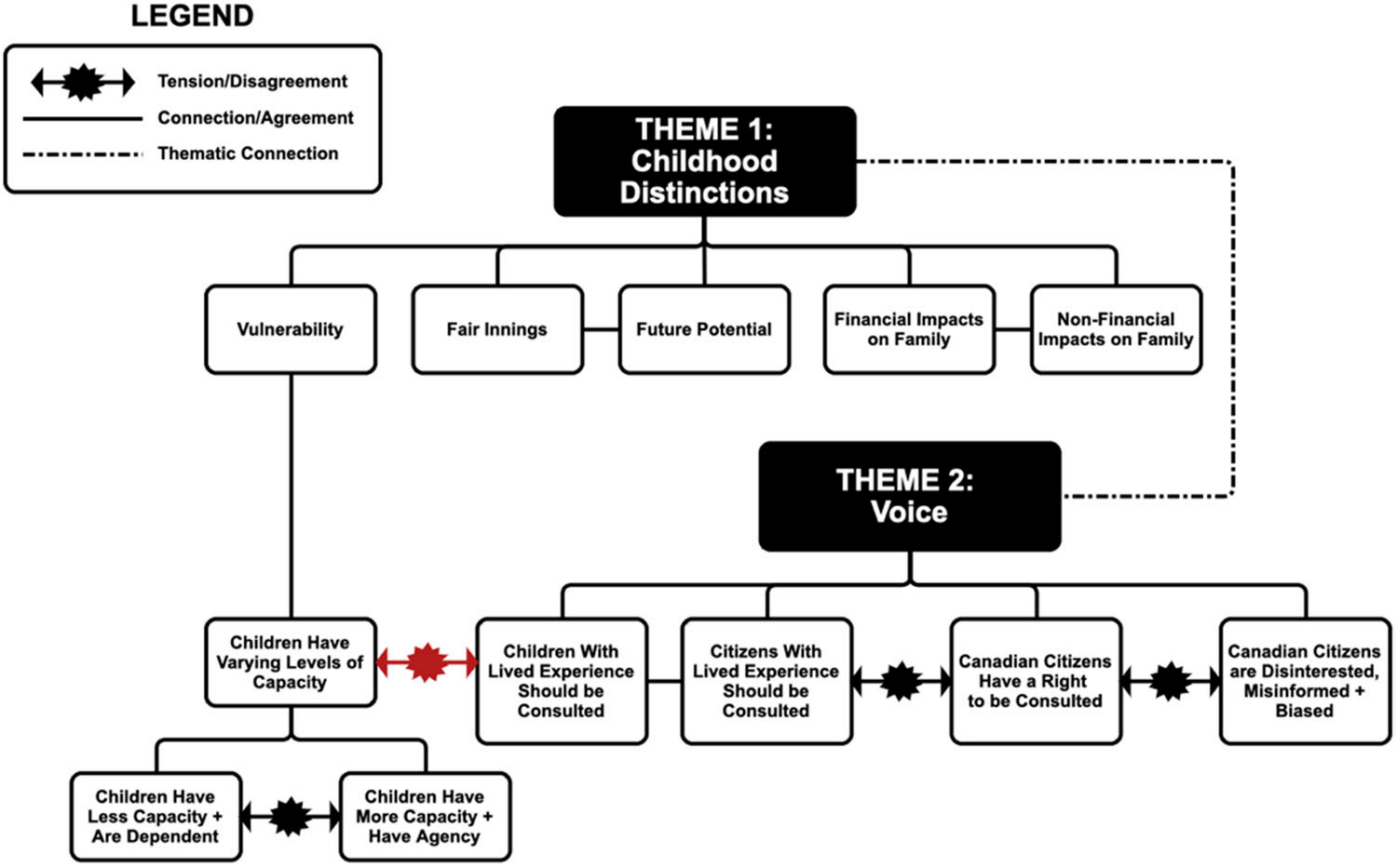
Illustration of relationship between two key themes: childhood distinctions and voice. Red‐coloured tension/disagreement denotes strong discordance.

Figure 2 shows the points of tension between subthemes within ‘Childhood Distinctions’ and ‘Voice’ and the connection to children's voices through the subtheme ‘Vulnerability’. A particularly rich discussion is highlighted through the subthemes ‘Children have varying levels of capacity’ and ‘Children with lived experience should be consulted’, within which there was strong discordance in participants' perspectives of children's involvement in clinical decision‐making. Participants dissented on the specific age when children might attain capacity, reflecting their personal experiences besides the varying ages of legal consent in provincial jurisdictions. Some panellists argued that some younger children do have the capacity to understand their care and should have the agency to voice their opinions. As one panellist expressed, ‘I've met a number of teenagers and even some kids around 9, 10 years old who would blow you away with their perspectives on the cancer treatment they're going through and the impact it's having on them’ (Panellist P2‐1).

**Figure 3:**
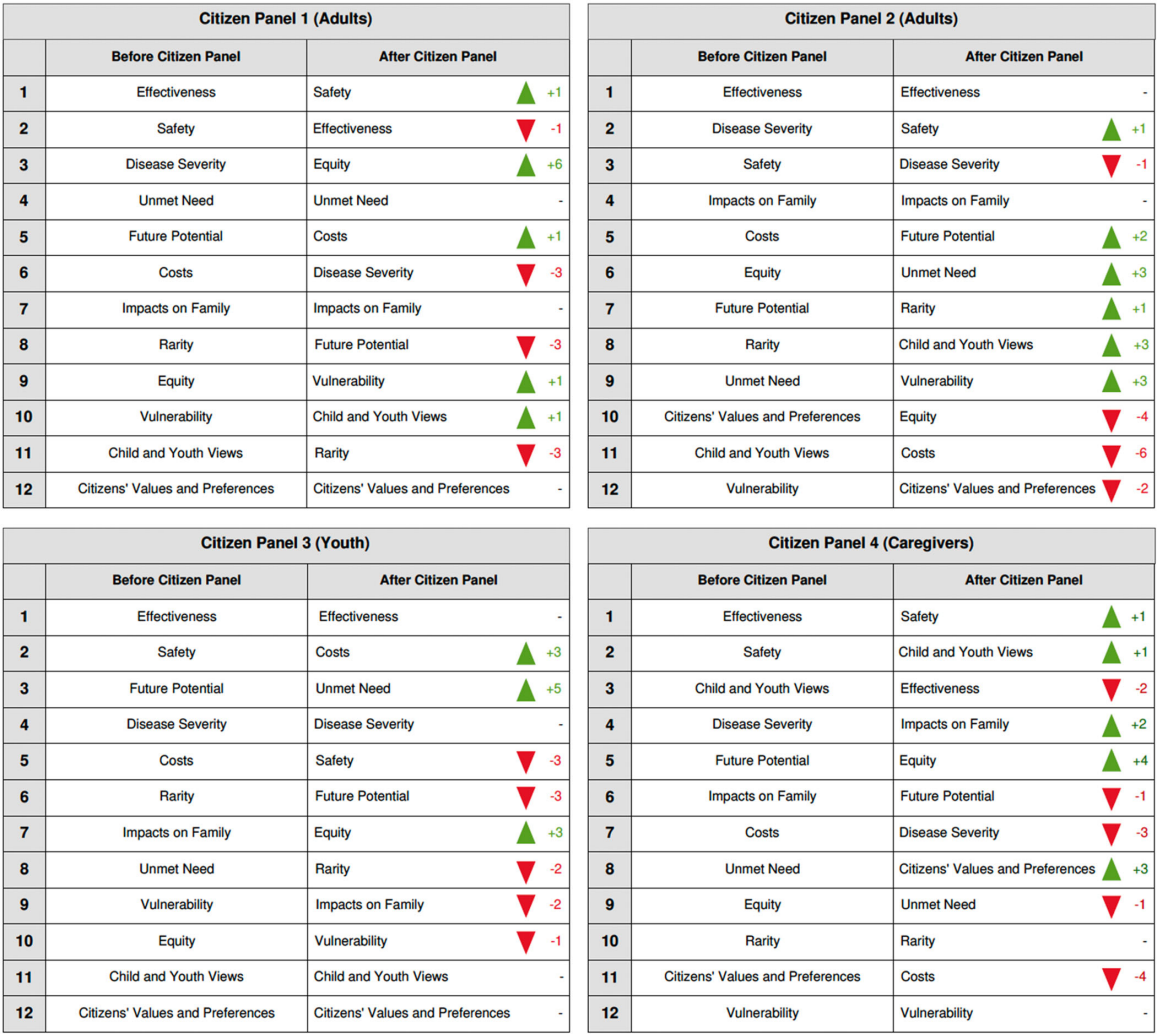
Panellists' pre‐ and postdeliberation ranking of health technology assessment (HTA) values

The dissent also manifested in the pre‐ and postdeliberation ranking of potential HTA values (Figure 3 below). The general public and youth panellists ranked the value of ‘children and youth views’ with low importance for HTA evaluations. As one panellist expressed, ‘honestly, I feel like the child and youth views can probably be taken off [the list of values] completely…’ (Panellist P3‐4). Conversely, caregivers not only ranked it highly in the predeliberation poll, third after effectiveness and safety but elevated it further to the second position in the postdeliberation poll.

### 3.6 Theme 3: One versus Many

There was a tension between views with humanitarian or utilitarian underpinnings, which emphasized opposing considerations about the individual and the collective as well as equality versus equity. These topics were most prominently articulated in the Youth panel.

Panellists with humanitarian views held that all Canadians deserve equal access to precision therapies in the healthcare system, regardless of their age, geographic location, the rarity of their disease, the price of the therapy and the jurisdictional authority over funding, the more so if the therapy has already been approved for marketing. They believed that no segment of the population should be prioritized over another. Evoking the sanctity of life one panellist argued, ‘…you can't put a price on someone's life. So definitely every single person deserves equal attention … or treatment. … we can't say this … person is more important than the other one’ (Panellist P2‐12).

Conversely, those expressing utilitarian values centred their perspectives around the notion of achieving the greatest good for the greatest number of people when allocating finite health system resources. Considering the rare nature of diseases that are treated with precision medicine and opportunity costs, a handful of panellists suggested diverting funding away from precision therapies for children and youth and towards medicines and treatments for more prevalent diseases, which may benefit a larger proportion of the population.

### 3.7 Theme 4: Health System Governance

Equity in access was at the heart of panellists' repeated expressions for the Canadian federal government to take a larger or even exclusive role in resolving unequal and variable funding for paediatric precision therapies between provinces. Some panellists even went so far as to suggest that the federal government claims jurisdiction over health care from the provinces. One panellist asserted they ‘…think what we really need to do in Canada … is … start looking at a constitutional amendment to put more medical in the federal rather than provincial realm’ (Panellist P2‐10). Federal government‐led funding mechanisms and coordination between provincial HTA bodies held appeal for many panellists, all with a view to a more integrated process of drug approval, regulation and funding evaluation.

Given the geographical vastness of Canada, to address inequities in access, panellists thought that government could consistently provide travel support for children facing socioeconomic constraints to participate in clinical trials or to obtain already‐funded treatments. In acknowledging the limits of the public purse, although, several panellists suggested patient/family out‐of‐pocket payment models such as co‐pay, or payment scaled to income, with provisos to safeguard equity of access.

A caregiver's ability, time and level of medical and legal literacy to advocate government on behalf of their children emerged as a unique factor contributing to inequity. One caregiver observed that ‘The steps, the policies, the bureaucracy … that's just another division that I can see forming is that the families that have the financial resources, but also like the [health] literacy resources to do this fighting will have, you know, maybe a better chance than the families who aren't doing this kind of active fighting and pushing’ (Panellist P4‐2).

Despite panellists' strong opinions on the role of government, and even among caregivers immersed in efforts to access paediatric precision drugs, there was a low level of awareness on federal and provincial HTA processes and their relationships to drug regulation and funding decisions at the provincial level.

### 3.8 Pre‐ and postdeliberation questionnaires on ranking of HTA values

The dynamic nature of panellists' debates across the four panels was captured by absolute and relative changes in how panellists ranked an a priori list of HTA‐relevant values (Figure 3) before and after deliberations. ‘Equity’ rose in importance in three of the four panels, while ‘Cost’ had the largest single drop amongst the values. Although panellists had expressed appreciation for being invited to provide their views, ‘Citizens' Values and Preferences’ ranked unchangingly near or at the bottom of three panels. The rankings of the caregiver panel stood apart from those of other panels, with ‘Child and Youth Views’ ranked among the highest values alongside therapeutic effectiveness and safety. Notably, by the end of their panel discussion, caregivers ranked ‘Child and Youth Views’ higher than ‘Effectiveness’.

## 4 DISCUSSION

Our study illustrates citizens' contributions to an ‘upstream’ phase of the HTA process, the development of a value framework, focussing on precision therapies for children and youth. We undertook a multi‐step approach to preparing an evidence‐informed brief and eliciting citizens' views through a method of consultation based on DPE. Panellists asserted that existing HTA frameworks could and should be revised to incorporate intrinsic, commonly held societal values associated with children and childhood and to better reflect child and family perspectives on the experiences of chronic illness. The biological, physiological and social distinctiveness of children compared to adults—particularly as encapsulated by the values of family impacts, vulnerability, ‘fair innings’ and future potential—was expressed as the principal reason to develop a child‐tailored HTA framework.

Although a child‐tailored value framework resonated strongly with our panellists, some important dissenting views emerged. Tensions founded on competing ethical views surfaced, such as the prioritization of health care for a population versus treatment for a few. When discussing vulnerability, panellists debated the delimitation of childhood, the balance of paternalism versus agency and autonomy for children, the reach of rights‐based protections for children as positive rights to access health technologies, and distinctions between the pursuit of equality versus equity. Many of the tensions among our panellists arose from dissenting views on age‐based prioritization of resources. Youths supported a neutral weighting of age, despite their own age proximity to the paediatric population under discussion.

Dissent was perhaps most pronounced in relation to the issue of representation, or ‘voice’, in HTA decision‐making. Panellists were divided as to the relative importance of those with lived experience. Further, whether and how to incorporate children's voices in evaluating technologies figured prominently in panellists' debates. Differences of opinion were evident in each panel, rather than sharply demarcated between caregivers and noncaregiver panels, as might be expected. Indeed, the most pointed debate about the legitimacy of children's voices arose in the Youth panel, none of whom were caregivers or parents. Finally, assessment rules for child health technologies distinct from those employed for adults were seen by some to clash with population‐level decision‐making in the context of Canada's universal public healthcare system.

In engaging the nonpatient public, including youth, we identified the wide range of values and the trade‐offs important to those who do not have a personal stake in a particular technology funding decision yet are subject to the financial and opportunity costs resulting from it. Values identified by our panellists as particularly salient to child precision health technologies—such as rarity, unmet need and family impacts—have varying resonance in other settings. A UK study using broad preference elicitation methods demonstrated that disease rarity was not prioritized by its citizen respondents.^48^ On the other hand, CADTH and INESSS have adapted to the changing pharmaceutical landscape by adopting unmet needs as a criterion for establishing therapeutic value.^49^, ^50^ In several European countries, values uncovered through expert deliberation that are not traditionally or formally used in decision‐making are framed as contextual or implementation factors. Similarly, in New Zealand's drug funding framework, the individual's connection to family and society is explicitly considered but not formally integrated into reimbursement decisions.^51^ As in our study, other research has identified hope as a potential value for extending HTA frameworks, particularly in the context of heightened evidentiary uncertainty and clinical risk.^52^

The potential for significant family impacts in paediatric contexts emerged as a key justification for extended or supplemental HTA frameworks focussed on child health.^53^, ^54^ Enabling a specific focus on the unique economic and noneconomic costs that accrue to caregivers and siblings of children with complex or chronic illnesses may allow a more adequate and comprehensive assessment of the value paediatric therapies.^30^, ^55^

We found that most panellists in our study, when prepared with evidence‐based resources, were eager and able to present views on evaluating paediatric precision therapies. The pre‐ and postdeliberation polls suggested that when panellists are given the chance to hear divergent views in a facilitated forum, they may make reasoned changes in judgement. Similarly, a Dutch study using DPE to examine citizens' preferences for inclusions in a social health insurance benefits package found that panellists could articulate a wide range of considerations on a complex topic, learn from the views of others and evolve their opinions through the course of deliberations.^56^ As with the panellists in our study, the Dutch panellists prioritized a number of criteria new to conventional HTA frameworks, including ‘societal side effects’, ‘taking away from people’ and ‘lifestyle’. Motivated by accelerating cancer therapy costs in Canada, DPE has been applied to explore the contentious issue of defunding cancer drugs in British Columbia^57^ and to generate recommendations for fairer, more sustainable cancer drug funding for the entire Canadian population.^58^ In the former study, panellists were concerned with defunding and its implications for equity of access and patient choice. In the latter, panellists felt that higher drug costs could be justified through improvements in patients' independence and mental health.

Emerging evidence suggests that DPE may result in improved health outcomes in certain settings.^59^, ^60^ In Canada, as elsewhere, the operationalization of DPE in HTA processes remains nascent. CADTH is actively reviewing the integration of deliberative processes into the appraisal stage of its HTA process,^39^ while INESSS has recently instituted a flexible evaluation framework that can be adapted to integrate public perspectives.^50^ Variability in defining the ‘public’ remains a key issue for scoping the specific role of participants, however.^61^, ^62^, ^63^ Patients and the public are often viewed interchangeably, and the wider public can be seen to include experts (e.g., clinicians, pharmacists), decision‐makers (e.g., government funders, third‐party payers), other vested stakeholders (e.g. patient/disease advocates, caregivers) or all citizens of a political jurisdiction. Our study lends support to the greater incorporation of children and youth as a legitimate source of public input for HTA framework development and decision‐making. However, implementation issues abound, including how to structure opportunities for participation appropriate to children's evolving developmental capabilities.

Our study has a number of strengths. It is, to our knowledge, the first structured, evidence‐informed deliberative process to engage the general public in value assessment framework development for paediatric health technologies, whether for precision therapies or otherwise. Incorporating the opinions of the nonpatient public in DPE is rare, and in HTA framework development, rarer still.^37^, ^60^, ^64^ By eliciting, distinguishing and reporting on the varied perspectives of citizens with and without lived experience of rare or hard‐to‐treat paediatric diseases, it provides a broad canvas of values relevant to child HTA, while also mapping out internal territories marked by the varied, and sometimes conflicting, perspectives of different types of ‘publics’. This variation is highlighted by the distinctive perspectives of our youth panellists, who are systematically underrepresented in HTA processes and in DPE more broadly. Finally, our deliberative engagement panels were informed by evidence briefs developed in consultation with a broad range of expert stakeholders, including relevant jurisdictional drug regulatory and HTA bodies, ensuring procedural rigour and optimising the potential for close alignment with real‐world policy needs and priorities.

A few limitations of our study are worth noting. Whereas most published experience with DPE to date has been premised on in‐person events, the COVID‐19 pandemic necessitated a wholly virtual format for panel meetings; panellists may have self‐selected to participate on the basis of comfort and technological capability with online meetings. Although a representative national sample was sought, there were no panellists from Canada's northern territories or the province of Newfoundland‐Labrador (largely rural regions with sparse populations and limited internet coverage outside capital cities). Consequently, some perspectives on access and equity issues were missed. Participants were predominantly English speakers, so our findings may retain less relevance for regions where different languages or cultures predominate, such as Québec and Nunavut. More generally, our findings should be cautiously applied to other jurisdictions internationally, each of which has its own admixture of sociopolitical and health system realities, with varied bearing on the regulation and reimbursement of health technologies for children and youth.

## 5 CONCLUSION

Citizens' perspectives afford rich insights into the range and multi‐dimensionality of criteria relevant to decision‐making about the allocation of scarce public funds for novel health technologies. They can serve as a key source of values to ground the design of normatively holistic and societally responsive value frameworks for adjudicating the worth of health technologies. Our study adds evidence on values relevant to priority‐setting for paediatric precision therapies that can inform the development of child‐focused tools and processes for HTA in Canada and comparable health systems internationally.

Future research is warranted to explore and specify the role of citizens in various aspects of HTA processes, including whose voice should be incorporated, how to balance diverse perspectives among varied groups and the optimal ways to operationalize citizen engagement in deliberative approaches to HTA. In the case of precision therapies, particularly for rare diseases, focussing on the distinctiveness of child health is essential, given the preponderance of genetically based diseases in childhood. How to balance the hopes and expectations of patients and caregivers for access to expensive but potential life‐altering therapies for relatively rare diseases against the opportunity costs borne by encompassing health systems remains a fundamental challenge. Rigorous methods to elicit, weigh and reconcile views from varied members of society, including children and youth, will prove an essential part of the response to this challenge if decisions about which health technologies we value are to reflect our shared and diverse social values.

## AUTHOR CONTRIBUTIONS

Cindy L. Gauvreau, Lisa Wight, Mathushan Subasri, Michael G. Wilson and Avram Denburg conceived and conducted the study. They drafted, edited and reviewed the Citizen Brief and this manuscript. Avram Denburg and Michael Wilson facilitated the citizen panels. Cindy L. Gauvreau, Lisa Wight, Mathushan Subasri prepared and analysed the data. Antonia Palmer, Robin Hayeems, Alysha Croker, Julia Abelson, Brent Fraser, Yvonne Bombard and Charlotte Moore Hepburn were members of the steering committee of this study and provided expert and technical advice. They assisted in key informant and panellist recruitment, reviewed and edited the Citizen Brief and reviewed and edited this manuscript. Antonia Palmer also prepared a video for presentation at the citizen panels.

## CONFLICT OF INTEREST

A. C. is a full‐time employee of Health Canada. The opinions expressed in this manuscript are the authors' own and do not reflect the views of Health Canada and may not be understood or quoted as being made on behalf of, or reflecting the position of, the organization with which the author is affiliated. The remaining authors declare no conflict of interest.

## ETHICS STATEMENT

The Hamilton Integrated Research Ethics Board (HiREB) provided approval for the citizen panels (HiREB project # 13‐369). The Hospital for Sick Children provided overall approval for this project (REB #1000069221).

## Supporting information

Supporting information.

## Data Availability

Data that support the findings of this study are available on request from the corresponding author. The data are not publicly available due to privacy or ethical restrictions.
