# Supplementary material for: Access to novel drugs and therapeutics for children and youth: Eliciting citizens' values to inform public funding decisions
Source: Health Expect. 2023 Jan 14;26(2):715–27. doi: 10.1111/hex.13697 (PMC10010086; doi:10.1111/hex.13697)
Supplement: Supplementary file 1 — Supporting information. [file HEX-26--s001.docx]

**Appendix 1: Complementary videos**

1. What is precision medicine? Cumming School of Medicine. University of Calgary, Calgary AB. 2021. <https://www.youtube.com/watch?v=6NdXsoo0j3I>
2. Intro to Health Technology Assessments. Find a Cure, United Kingdom. 2021. <https://www.youtube.com/watch?v=UpzP2VfbEUA>
3. CADTH Drug Reimbursement Reviews. CADTH, Ottawa, ON. 2021. <https://www.youtube.com/watch?v=TqC8f44nny4>
4. 19-year-old not covered for costly treatment for rare disease. CBC News, Canadian Broadcasting Corporation. Toronto, ON 2021. .<https://www.youtube.com/watch?v=zlvyrLLMVQU>

**Appendix 2. Codebook**

| **Category** | **Code** | **References** | **Coverage (%)** |
| --- | --- | --- | --- |
| **Costs** | Costs discussed generally | 42 | 22.89 |
|  | Cost as a barrier to access | 32 | 21.15 |
|  | Cost effectiveness should be considered in HTA decisions | 9 | 5.18 |
|  | Costs should be considered in HTA decisions | 9 | 10.39 |
|  | Innovative funding | 39 | 22.71 |
|  | Opportunity costs | 32 | 18.87 |
| **Disease Severity** | Disease severity discussed generally | 7 | 2.77 |
|  | Disease severity is an important value | 12 | 5.43 |
| **Effectiveness** | Effectiveness discussed generally | 56 | 29.75 |
|  | Diminishment of effectiveness should be considered in HTA decisions | 38 | 24.36 |
|  | Effectiveness is an important value | 29 | 13.07 |
|  | Uncertainty of data on effectiveness should be considered in HTA decisions | 36 | 30.77 |
| **Equity** | Equity discussed generally | 34 | 22.35 |
|  | Geography | 60 | 37.93 |
|  | Income | 35 | 21.37 |
|  | Other inequities | 14 | 12.16 |
| **Fair Innings** | Fair innings discussed generally | 38 | 46.60 |
|  | Children have their lives ahead of them and deserve a chance | 38 | 19.54 |
| **Future Potential** | Future potential discussed generally | 22 | 15.58 |
|  | Children's future contributions to society | 40 | 22.62 |
| **Future Research and Treatments** | Future research and treatments discussed generally | 28 | 30.60 |
|  | Existing therapies could be bridges to new therapies | 15 | 22.72 |
| **Healthcare System** | Healthcare system discussed generally | 30 | 13.86 |
|  | Canada could become a world leader in HTA | 4 | 5.20 |
|  | Decreased burden on healthcare system in future | 17 | 8.37 |
|  | Federal government should be responsible for healthcare | 29 | 22.29 |
|  | Increased public and government awareness of HTA | 24 | 21.82 |
|  | Integrated care | 14 | 14.00 |
|  | Limited health budget a barrier to access | 11 | 10.52 |
| **Hope** | Hope discussed generally | 54 | 28.60 |
| **Humanitarian Perspective** | Humanitarian perspective discussed generally | 48 | 23.32 |
| **Impacts on Family** | Impacts on family discussed generally | 83 | 45.58 |
|  | Parents want best outcome for their children | 18 | 11.09 |
| **Importance or Promise of Precision Therapies** | Importance or promise of precision therapies discussed generally | 25 | 10.73 |
| **Misunderstanding or Confusion** | Misunderstanding or confusion discussed generally | 28 | 21.26 |
| **Pharmaceutical Companies** | Pharmaceutical companies discussed generally | 32 | 28.44 |
|  | Excessive profit-taking | 10 | 10.48 |
|  | Pharmaceutical companies should reduce costs | 3 | 2.28 |
| **Public Input** | Public input discussed generally | 28 | 23.95 |
|  | Caregivers with lived experience should be consulted | 20 | 20.24 |
|  | Children with lived experience should be consulted | 39 | 32.35 |
|  | Citizens with lived experience should be consulted | 20 | 21.99 |
|  | Citizens' values and preferences are less important | 32 | 18.58 |
|  | Citizens' values and preferences could be important | 19 | 11.64 |
|  | Experts should be consulted | 8 | 8.26 |
|  | Citizens are hesitant to give input | 27 | 9.07 |
| **Ranking Values** | Ranking values discussed generally | 49 | 31.96 |
| **Rarity** | Rarity discussed generally | 42 | 31.25 |
| **Safety** | Safety discussed generally | 15 | 18.49 |
|  | Safety is an important value | 18 | 8.84 |
|  | Uncertainty of or risks associated with precision therapies | 17 | 11.12 |
| **Understanding Values** | Understanding values discussed generally | 16 | 17.72 |
|  | All values are important | 11 | 5.84 |
|  | Inadequacy of list or confusion with definitions | 27 | 18.71 |
|  | Values are related to one another | 6 | 12.48 |
| **Uniqueness of Children** | Uniqueness of children discussed generally | 26 | 40.43 |
|  | Children are not special or unique | 19 | 24.75 |
|  | Children are special or unique | 77 | 36.95 |
| **Unmet Need** | Unmet need discussed generally | 19 | 16.14 |
|  | Availability and effectiveness of alternative treatments should be considered in HTA decisions | 14 | 12.65 |
| **Utilitarian Perspective** | Utilitarian perspective discussed generally | 30 | 12.67 |
| **Vulnerability** | Vulnerability discussed generally | 24 | 16.97 |
|  | Children have or should have agency within family | 6 | 4.67 |
|  | Children have varying levels of capacity | 46 | 26.73 |
|  | Children depend on parents to make decisions | 39 | 26.26 |
